# Genome-wide identification and expression analysis of the bZIP transcription factor family genes in response to abiotic stress in *Nicotiana tabacum* L.

**DOI:** 10.1186/s12864-022-08547-z

**Published:** 2022-04-22

**Authors:** Lili Duan, Zejun Mo, Yue Fan, Kuiyin Li, Mingfang Yang, Dongcheng Li, Yuzhou Ke, Qian Zhang, Feiyan Wang, Yu Fan, Renxiang Liu

**Affiliations:** 1 College of Agriculture, Guizhou University, Guiyang, 550025 People’s Republic of China; 2 Guizhou Key Laboratory for Tobacco Quality Research, Guizhou University, Guiyang, 550025 People’s Republic of China; 3 College of Tobacco, Guizhou University, Guiyang, 550025 People’s Republic of China; 4 College of Food Science and Engineering, Xinjiang Institute of Technology, Aksu, 843100 People’s Republic of China; 5 School of Food and Biological Engineering, Chengdu University, Chengdu, 610106 People’s Republic of China

**Keywords:** bZIP family, *Nicotiana tabacum* L., Gene expression patterns, Abiotic stress

## Abstract

**Background:**

The basic leucine zipper (bZIP) transcription factor (TF) is one of the largest families of transcription factors (TFs). It is widely distributed and highly conserved in animals, plants, and microorganisms. Previous studies have shown that the bZIP TF family is involved in plant growth, development, and stress responses. The bZIP family has been studied in many plants; however, there is little research on the *bZIP* gene family in tobacco.

**Results:**

In this study, 77 *bZIP*s were identified in tobacco and named *NtbZIP01* through to *NtbZIP77*. These 77 genes were then divided into eleven subfamilies according to their homology with *Arabidopsis thaliana*. *NtbZIP*s were unevenly distributed across twenty-two tobacco chromosomes, and we found sixteen pairs of segmental duplication. We further studied the collinearity between these genes and related genes of six other species. Quantitative real-time polymerase chain reaction analysis identified that expression patterns of *bZIP*s differed, including in different organs and under various abiotic stresses. *NtbZIP49* might be important in the development of flowers and fruits; *NtbZIP18* might be an important regulator in abiotic stress.

**Conclusions:**

In this study, the structures and functions of the *bZIP* family in tobacco were systematically explored. Many *bZIP*s may play vital roles in the regulation of organ development, growth, and responses to abiotic stresses. This research has great significance for the functional characterisation of the tobacco bZIP family and our understanding of the bZIP family in higher plants.

**Supplementary Information:**

The online version contains supplementary material available at 10.1186/s12864-022-08547-z.

## Background

Transcription factors (TFs) are proteins that combine with specific short sequences of DNA to regulate the expression of genes [1, 2]. Important regulatory factors, TFs bind to the promoter regions of target genes and activate or inhibit their transcription. Among the TF families, the basic leucine zipper (bZIP) TF family is the most widely distributed and conserved in the domain of eukaryotes, found in humans, plants, animals, and microorganisms [3, 4].

The bZIP TF family is named after the bZIP domain [5]. The bZIP protein sequence contains 2 conserved bZIP domains with a total length of ~ 60–80 amino acids. The first is a highly conserved domain containing an N- × 7-R/K sequence, which consists of approximately 16 amino acid residues and functions in localization and specific binding to DNA [6, 7]. The second is a relatively poorly conserved leucine zipper region characterised by leucine in the last position of every seven amino acids, and hydrophobic residues at positions 3 and 4. The α-helix is a necessary structure of dimer required for bZIP proteins to bind to DNA [8–10]. The core sequence of bZIP TFs is the cis acting element of ACGT, e.g., as found in the G-box (CACGTG), C-box (GACGTC), the abscisic acid (ABA)-responsive element, and the A-box (ACGTA) [11–14].

Research into the bZIP family in different species will bring new insights into its evolution and function. Previous studies have shown that the bZIP TF family is involved in the regulation of genes involved in organ and tissue differentiation [15–18], seed storage [19, 20], pathogen defence [21], the unfolded protein response [22–24], photomorphogenesis and light signal regulation [25, 26], hormone and sugar signaling processes [27, 28], and responses to abiotic stress [3, 29, 30]. Different subfamilies regulate different biological processes. Studies have shown that *AtbZIP30* is involved in regulating cell development, organ development, hormone homeostasis, and meristematic processes [16]. Some bZIP TFs are activated under abiotic stress; they can combine with the promoter regions of key genes in signaling pathways to regulate their transcription levels and, in turn, plant resistance to stress. For example, research has identified that the expression of *EcbZIP60*, a gene homologous to *AtbZIP60*, is upregulated in response to drought and salt stresses [25]. In *Arabidopsis thaliana*, HY5, a member of the bZIP TF family, has been shown to inhibit hypocotyl growth and lateral root development, and promote pigment accumulation in a light-dependent manner [15]. *OsbZIP12* is a member of the bZIP family in rice that has been shown to be involved in ABA signal transduction and regulation of drought resistance [26]. *RsbZIP031* and *RsbZIP059* in radish were significantly downregulated under high temperature and salt stress, respectively [27]. In pineapple root tissues, *AcobZIP24* has a significant response to salt, drought, cold, and heat stresses at 24 and 48 h [28]. These examples illustrate the response of bZIP TFs in plants to various abiotic stresses.

*Nicotiana tabacum* L. is a tobacco plant of the Solanaceae. Tobacco is an important cash crop in agriculture, and is widely cultivated in more than 120 countries around the world [31–34]. Besides *A. thaliana*, tobacco is also often used as a model plant for the study of biological mechanisms because of the advantages conferred by its short growth time, disease susceptibility, and easy genetic transformation [35]. In addition, there is new interest in tobacco for its potential in biopharmaceuticals and biofuel production [36, 37]. Thriving in the natural environment, tobacco is stressed by salt, drought, cold, heat, and flooding, which seriously impact its growth, yield, and quality [17, 18, 38–40]. The bZIP family has been comprehensively identified and studied, with 75 *bZIP*s identified in *A. thaliana* [7, 8], 89 in rice [4], 131 in soybean [9], 55 in grape [41], 90 in potato [42], and 52 in *Carthamus tinctorius* [43], with further *bZIP* genes identified in other plant species [44–46]. However, research into the bZIP family of tobacco is lacking. The development of the Tobacco Genome Initiative has provided many opportunities for the study of genome-wide gene families in tobacco [19, 20]. Furthermore, the emergence of next-generation sequencing technology enabled the problems of high cost and low yield to be overcome, making the development of tobacco genome sequencing more feasible [8, 9, 47–49]. In this study, we identified *bZIP*s from *Nicotiana tabacum* L., and studied their gene structure, chromosomal location, duplication, gene expression patterns, and responses to 6 abiotic stresses. We also studied the changes in enzyme activity occurring in plants under stress. In addition, an analysis of the homology between *bZIP*s in *A. thaliana*, *Oryza sativa*, *Fagopyrum tataricum*, *Solanum lycopersicum*, *Hordeum vulgare*, *Vitis vinifera, Solanum tuberosum* and tobacco was conducted. This study provides a theoretical basis for the mechanisms of stress resistance in tobacco and other species, and could open new research avenues in the breeding of high-quality tobacco varieties.

## Results

### Identification of *NtbZIP*s genes in tobacco

In this study, we identified 77 *NtbZIP*s in the tobacco database using two BLAST methods, and named them *NtbZIP01* through to *NtbZIP77* according to their chromosomal locations (from top to bottom) (Additional file 1: Table S1). The physical properties and basic characteristics of the predicted 77 *NtbZIP*s were analysed, including the coding sequence length (CDS), protein molecular weight (Mw), isoelectric point (pI), and predicted subcellular location (W) (Additional file 1: Table S1). The protein length of the predicted 77 NtbZIPs ranged from 85 (NtbZIP43) to 973 amino acids (NtbZIP76), with an average of 369 amino acids. The lowest Mw was 10.11 kDa (NtbZIP43), whilst the highest was 104.64 kDa (NtbZIP76); the average Mw was 40.77 kDa. The pI ranged from 4.17 (NtbZIP32) to 10.19 (NtbZIP52), with a mean of 7.11. Furthermore, analysis of predicted subcellular locations indicated that 64 NtbZIPs were located to the nuclear, 8 to chloroplasts, 3 (NtbZIP21, NtbZIP43, NtbZIP68) to the cytoplasm, 1 (NtbZIP02) to vacuole, and 1 (NtbZIP51) to the endoplasmic reticulum. The ratio of *NtbZIP*s to total genes in the tobacco genome was approximately 0.10%, similar to Chinese jujube (0.13%) [45], but lower than *Chenopodium quinoa* (0.16%) [41], castor bean (0.19%) [50], *A. thaliana* (0.20%) [8], and banana (0.35%) [51].

### Phylogenetic analysis and classification of *bZIP*s in tobacco

**Fig. 1:**
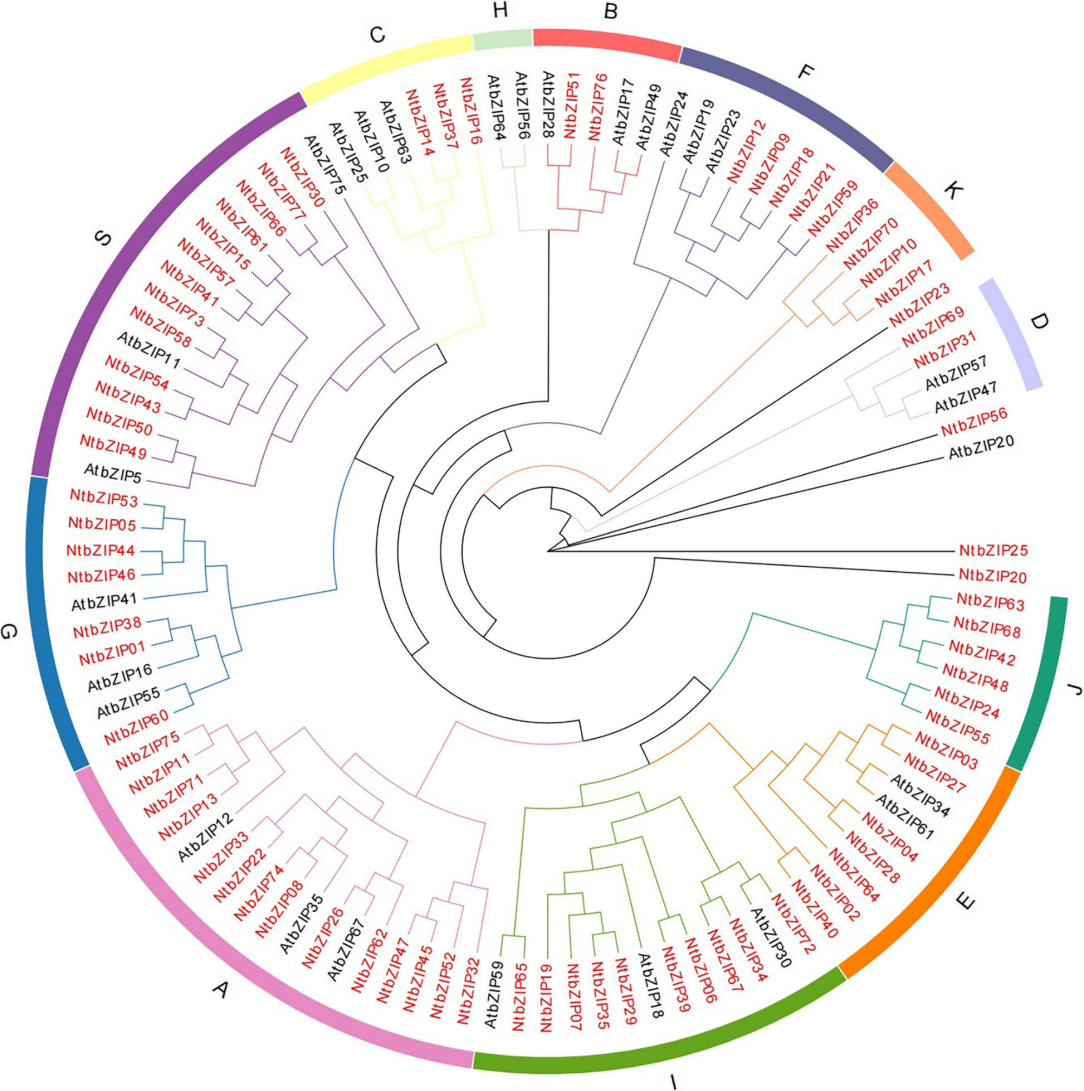
Unrooted phylogenetic tree showing relationships between the bZIP domains of *Nicotiana tabacum* L.and *Arabidopsis thaliana*. The phylogenetic tree was derived using the neighbour-joining method in MEGA X. The tree shows the 11 phylogenetic subfamilies (red). bZIP proteins from *Arabidopsis* are indicated by the prefix ‘At’. bZIP: basic leucine zipper; MEGA: Molecular Evolutionary Genetics Analysis

To study the evolutionary relationship between the *bZIP* families of *A. thaliana* and tobacco*,* a phylogenetic tree was constructed based on the amino acid sequences of bZIPs in *A. thaliana* and tobacco (Fig. 1; Additional file 1: Table S1). We used the method proposed by Jakoby et al. [8] to construct an unrooted neighbour-joining tree for classification. The 77 identified *NtbZIP*s were clustered into 11 subfamilies (A-G, I, J, K, S) and four ‘orphan genes’. Among these 11 subfamilies, 9 (A-G, I, and S) were similar to those found in *A. thaliana*, and the subfamilies were named to be consistent with *A. thaliana* [8]. We also identified 2 new subfamilies, J and K. These results indicated that the *bZIP* family is conserved in the evolutionary history of tobacco; these conserved subfamilies may play an important role in the process of evolution. The A subfamily was the largest subfamily identified, with 14 *NtbZIP* members; the S subfamily was second largest, containing 13 *NtbZIP*s. The smallest subfamilies were the B and D subfamilies, each containing 2 genes. The C, E, F, G, and I subfamilies had 3, 7, 5, 7, and 10 members, respectively. Subfamily H from *A. thaliana* (consisting of only *AtbZIP56* and *AtbZIP64*), contained no *NtbZIP* members, indicating that this group was lost during tobacco evolution (Fig. 1). Conversely, subfamilies J and K contained only *NtbZIP*s (6 and 4, respectively), suggesting that this may be a unique evolutionary direction in tobacco. Aside from the 11 subfamilies, 4 genes (*NtbZIP20*, *NtbZIP23*, *NtbZIP25*, and *NtbZIP56*) were not divided into any subfamily, which suggested that these 4 genes may have unique functions. The phylogenetic trees of *A. thaliana* and tobacco identified some *NtbZIPs* that were closely linked to *AtbZIPs*; these genes could potentially be homologous with the *AtbZIP*s and have similar physiological functions.

### Gene structure and motif analysis of the *NtbZIP* genes

**Fig. 2:**
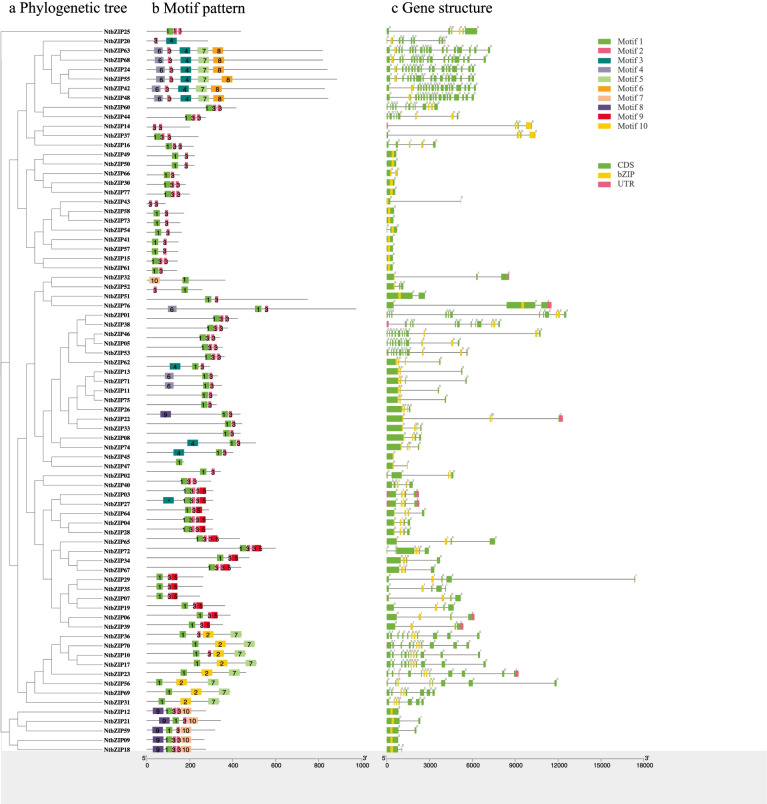
Phylogenetic relationships, gene structure analysis, and motif distributions of *Nicotiana tabacum* L. *bZIP*s. bZIP: basic leucine zipper. **a** Phylogenetic tree constructed by the neighbour-joining method, with 1000 replicates on each node. **b** Amino acid motifs in the bZIP proteins (1–10) are represented by coloured boxes. Black lines indicate relative protein lengths. **c** Exons and introns are indicated by green rectangles, and grey lines, respectively

By comparing the corresponding genomic DNA sequences, all *NtbZIP*s were further analysed to better understand the structural characteristics, including the number and sistruction of exons and introns (Fig. 2a,b). The numbers of introns present in the *NtbZIP*s ranged from 0 to 17 (Additional files 1: Tables S1). In total, 13 *NtbZIP* genes (16.88%) had no intron, 8 (10.39%) had 1 intron, 6 (7.79%) had 2 introns, 22 (28.57%) had 3 introns, 4 (5.19%) had 4 introns, 2 (2.60%) had 5 introns, 1 (1.30%) had 6 introns, 3 (3.90%) had 7 introns, 1 (1.30%) had 8 introns, 4 (5.19%) had 11 introns, 1 (1.30%) had 16 introns, 5 (6.49%) had 17 introns, respectively. The genes with no intron belonged to the A, F, and S subfamilies, and predominantly in the S subfamily. The 22 *NtbZIP*s with 3 introns accounted for the largest proportion of genes (28.57%). *NtbZIP24*, *42*, *48*, *63*, and *68* had the most number of introns, 17, and all of these genes belonged to the J subfamily. In general, genes in the same subgroup had a similar structure. For example, all genes in the E subfamily (except *NtbZIP40*) had 4 exons and 3 introns; all genes in the J subfamily (except *NtbZIP55*) had 18 exons and 17 introns.

In order to study the characteristic regions of the NtbZIP proteins, we used the MEME online tool to predict conserved motifs. A total of 10 conserved motifs (motifs 1–10) were identified from the 77 NtbZIP proteins (Fig. 2c, Additional file 2: S2). The length of the motifs ranged from 15 (motif 3) to 50 (motif 2, 4, 7, 8, 9, 10) (Additional file 2: S2). Motif 1 and 3 were widely distributed in the majority of NtbZIPs and these 2 motifs were very closely positioned in proteins, suggesting that the two motifs may play an important role in particular functions. As expected, members from the same subfamily shared similar motif compositions, particularly between a few close members. For example, members of the J subfamily had the same motifs: 3, 4, 6, 7, and 8. In subfamily S, apart from *NtbZIP43* which only contained motif 3, the rest of the members included both motifs 1 and 3; in addition, motif 9 was found in all the members of subfamily F. Furthermore, we also found that some motifs only appeared in certain subfamilies; e.g., motif 8 only appeared in subfamily J, indicating that this motif is specific to this subfamily. Analysis also identified that some of the 10 motifs only occurred in particular orders. In the J subfamily, except for where motifs were repeated, the motifs only appeared in a specific order: 6–3-4–7-8. Similarly, this also occurred in the F subfamily, in which the order of motifs was 9–1-3–10. In summary, the genes that in the same subfamily shared similar gene structure ana motif composition, providing further support for the phylogenetic analysis and subfamily classifications.

### Chromosome location and duplication of *bZIP*s in tobacco

**Fig. 3:**
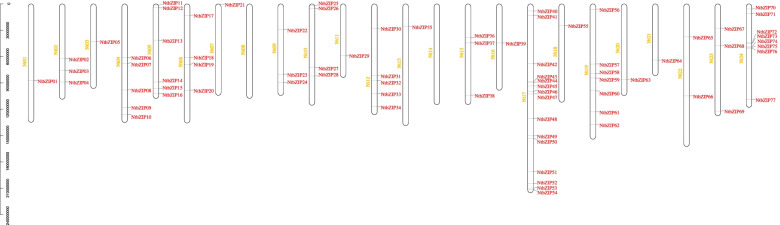
Schematic representation of the chromosomal distribution of the *Nicotiana tabacum* L. *bZIP*s. Vertical bars represent the tobacco chromosomes. Chromosome number is indicated to the left of each chromosome (yellow). bZIP: basic leucine zipper

On the basis of the tobacco genome sequence, we constructed a map of the physical positions of the *NtbZIP*s (Fig. 3). As shown in Fig. 3, all the identified 77 *NtbZIP*s were located on tobacco chromosomes, and the nomenclature of the genes was guided by their physical positions on tobacco Chr1 to Chr24. Aside from Chr8 and Chr14, on which no *NtbZIP* was distributed, the number of genes distributed on the remaining chromosomes ranged from 1 to 15. Chr17 contained the largest number of *NtbZIP*s (15 genes, 19.48%), followed by Chr24 (8 genes, 10.39%), Chr19 (7 genes, 9.09%) and Chr5 (6 genes, 7.79%). Chr1, Chr3, Chr7, Chr11, Chr13, Chr16, Chr18, Chr20, and Chr21 included just 1 gene (~ 1.30%), Chr22 contained 2 *NtbZIP*s (~ 2.60%), and Chr2, Chr9, Chr15, and Chr23 each contained 3 *NtbZIP*s (~ 3.90%). Chr6 and Chr10 had 4 (~ 5.19%) *NtbZIP*s, whilst Chr4 and Chr12 all included 5 *NtbZIP*s (6.49%). There was a correlation between chromosome size and the number of genes on chromosome. Such as, the largest chromosome (Chr17) contains 15 genes, and the smallest chromosome (Chr21) contains only one gene.

**Fig. 4:**
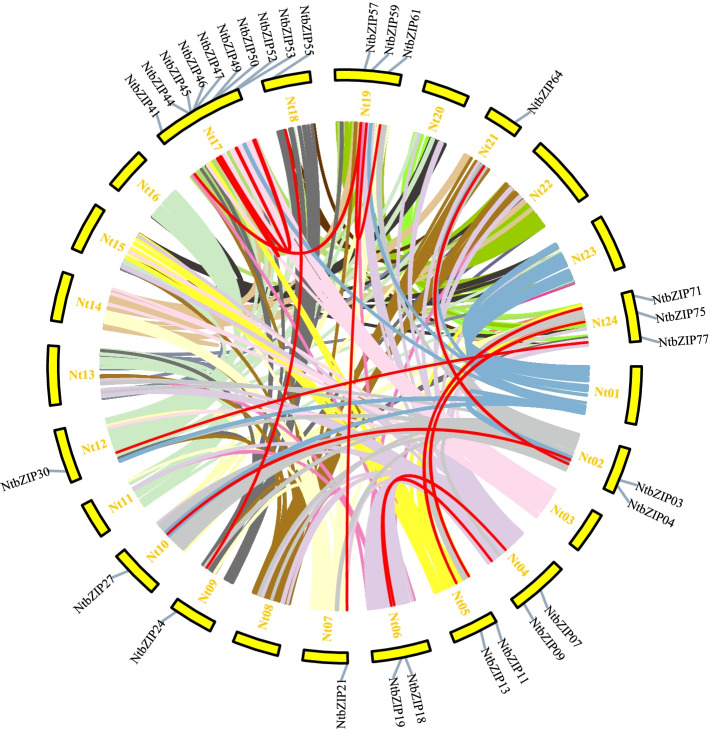
Schematic representation of the chromosomal distribution and interchromosomal relationships of *Nicotiana tabacum* L. *bZIP*s. Coloured lines indicate all synteny blocks in the *N. tabacum* genome; red lines indicate duplicated *bZIP* gene pairs. Chromosome number is indicated at the bottom of each chromosome. bZIP: basic leucine zipper

Furthermore, 16 pairs of segmental duplication were observed in the *NtbZIP*s (Fig. 4, Additional file 3: Table S3) and no tandem duplication was observed. Twenty-nine (37.66%) paralogs were identified in the *NtbZIP* family, indicating an evolutionary relationship between these *bZIP* members (Fig. 4). The paralogous *NtbZIP*s were unevenly distributed between 13 tobacco linkage groups (LGs) (Fig. 4). LG17 contained the most *NtbZIP*s (9), whilst LG7, LG9, LG10, LG12, LG18, and LG21 had the least (1). LG2, LG4, LG5, and LG6 each contained 2 genes, and there were 3 genes in LG19 and LG24. We also found that the 16 pairs of paired genes were linked within their subfamilies of S, A, F, G, and I.

### Synteny analysis of *NtbZIP*s

**Fig. 5:**
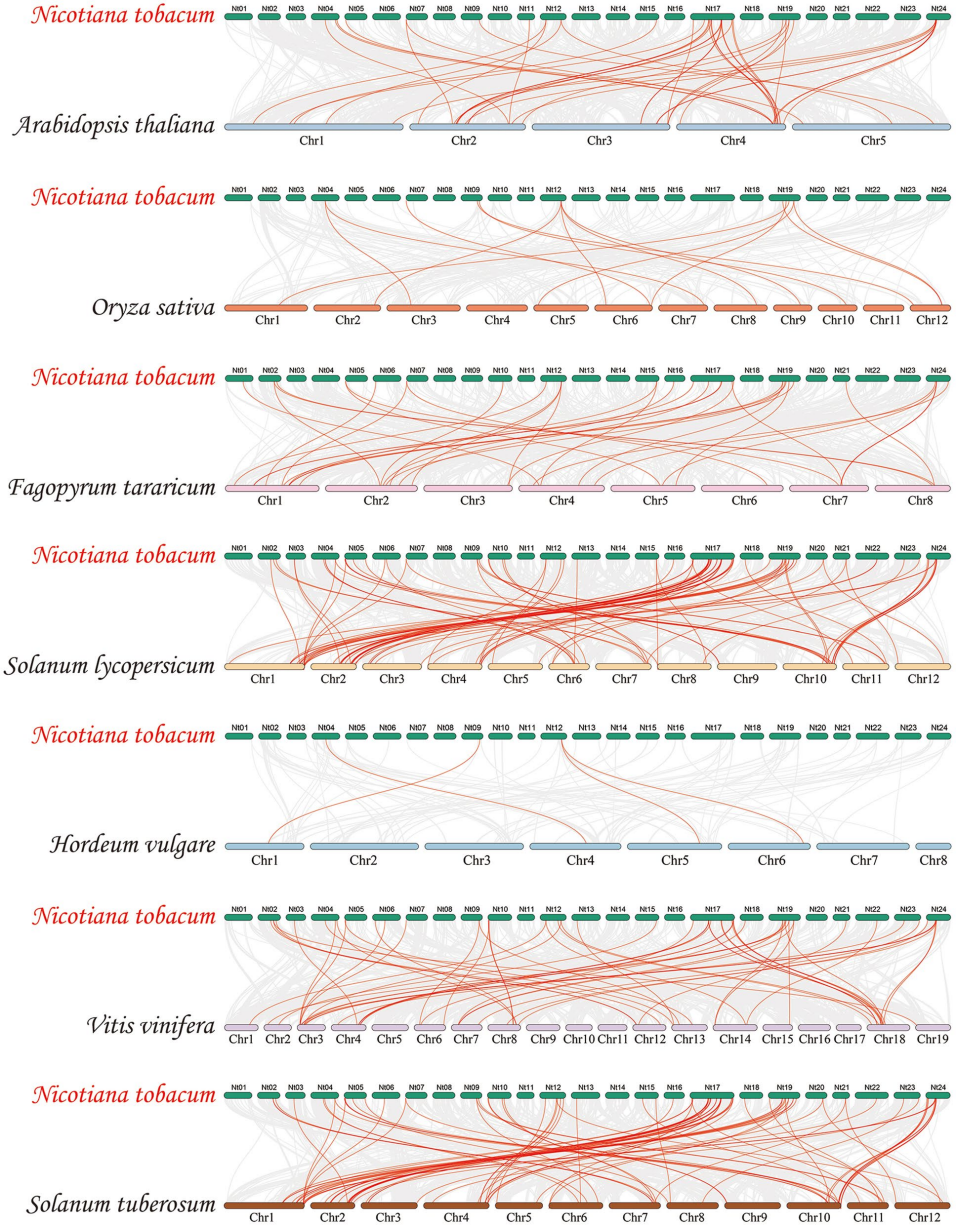
Synteny analyses of the *bZIP*s between *Nicotiana tabacum* and 7 representative plant species (*Arabidopsis thaliana*, *Oryza sativa*, *Fagopyrum tataricum*, *Solanum lycopersicum*, *Hordeum vulgare*, *Vitis vinifera* and *Solanum tuberosum*). Grey lines on the background indicate the collinear blocks in *N. tabacum* and other plant genomes; red lines highlight the syntenic *N. tabacum bZIP* gene pairs. bZIP: basic leucine zipper. Outer panel: An unrooted phylogenetic tree constructed using Geneious R11 with the neighbour-joining method. Inner panel: Distribution of the conserved motifs in bZIP proteins. The differently coloured boxes represent different motifs and their positions in each bZIP protein sequence. The sequence information for each motif is provided in Additional file 2: Table S2

To analyse the evolutionary relationship of the tobacco bZIP family, we constructed 7 synteny maps, comparing tobacco with six representative species, including *A. thaliana*, *S. lycopersicum*, *F. tataricum*, *O. sativa*, *H. vulgare*, *V. vinifera* and *S.tuberosum* (Fig. 5, Additional file 4: Table S4). In total, 68 *NtbZIP*s displayed a syntenic relationship across the 7 species, indicating that the tobacco *bZIP*s were highly conserved on the corresponding chromosomes in the process of evolution. The largest number of *NtbZIP*s displaying a syntenic relationship occurred when comparing the tobacco genes to *S.tuberosum* (60),, followed by *S. lycopersicum* (59), *V. vinifera* (45), *A. thaliana* (29), *F. tataricum* (27), *O. sativa* (9), and lastly *H. vulgare* (3) (Additional file 4: Table S4). The number of orthologous pairs between the other 7 species analysed (*A. thaliana*, *O. sativa*, *F. tataricum*, *S. lycopersicum*, *H. vulgare*, *V. vinifera* and *S.tuberosum*) was 44, 14, 35, 97, 4, 59 and 92, respectively. These result illustrated that the genetic relationship of *NtbZIP* genes, *SlbZIP* genes and *StbZIP* genes were close, which was reasonable because they are all Solanaceae plants. Several *NtbZIP*s were associated with at least 6 syntenic gene pairs: *NtbZIP07*, *NtbZIP21*, *NtbZIP24*, *NtbZIP30*, *NtbZIP33*, *NtbZIP38*, *NtbZIP41*, *NtbZIP43*, *NtbZIP49*, *NtbZIP50*, *NtbZIP52*, *NtbZIP57*, *NtbZIP59*, *NtbZIP60*, and *NtbZIP73*, indicating that these genes may have played an important role in the evolution of the *bZIP* family in tobacco. Furthermore, *NtbZIP33* was found in tobacco and all of the other 7 species, indicating that this orthologous pair appeared earlier than ancestral differentiation, and that *NtbZIP33* may have played a vital role in the evolution of the tobacco *bZIP* family.

### Expression patterns of *NtbZIP*s in different plant tissues and organs

**Fig. 6:**
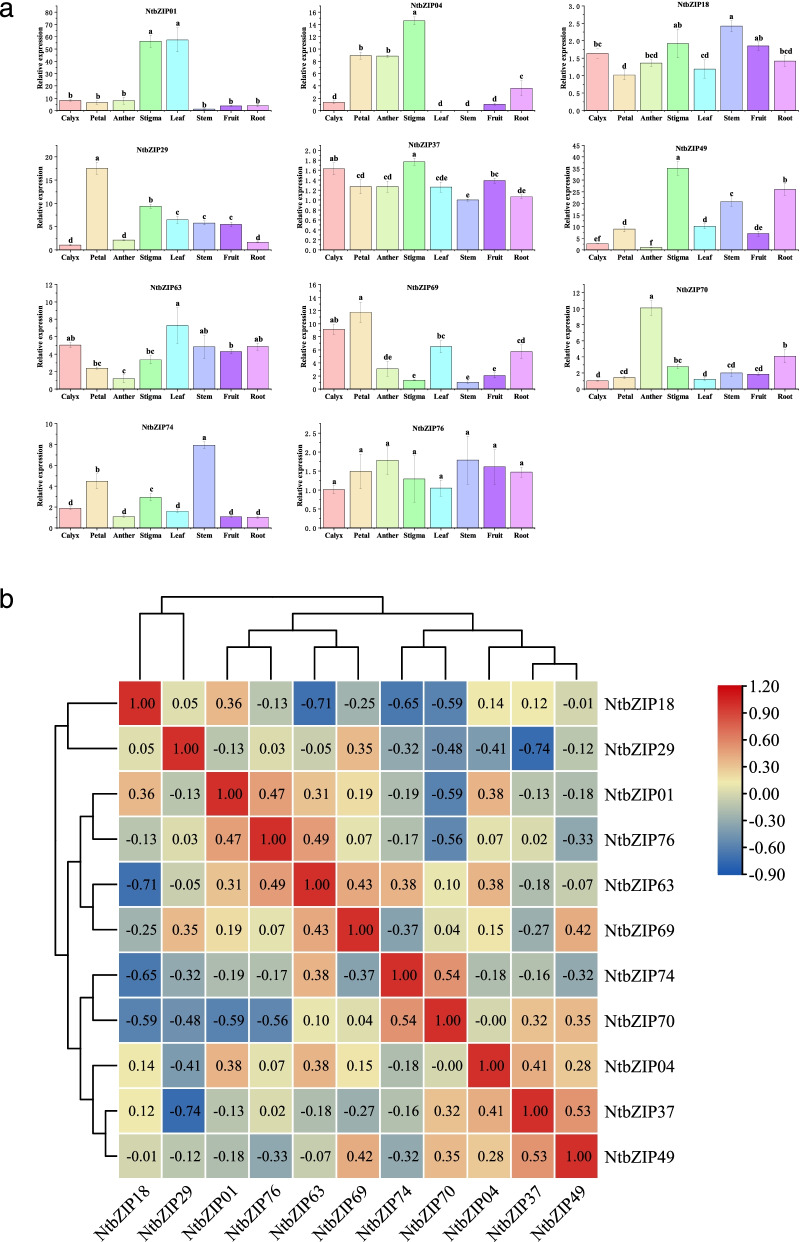
Tissue-specific gene expression of 11 *Nicotiana tabacum* L. *bZIP*s and the correlation between their expression patterns. bZIP: basic leucine zipper; qRT-PCR: Real-time quantitative PCR. **a** Expression patterns of 11 *Nicotiana tabacum* L. *bZIP*s in the calyx, petal, anther, stigma, leaf, stem, fruit, and root organs, as examined by qRT-PCR. Error bars represent standard error. Lowercase letters above bars indicate a significant difference (*P* < 0.05, least significant difference test) among the treatments. **b** Positive number: positively correlated; negative number: negatively correlated. Red numbers indicate a significant correlation at the 0.05 level

To investigate the physiological roles of the *NtbZIP*s, we selected 11 *NtbZIP*s from different subfamilies to study gene expression patterns. The expression levels of these *NtbZIP*s in eight tissues (calyx, petal, anther, stigma, leaf, stem, fruit, and root) were evaluated by quantitative real-time polymerase chain reaction (qRT-PCR) (Fig. 6a). The different expression patterns found for each gene in different organs indicate that *NtbZIP*s have different functions in tobacco. Most of the genes were expressed in all organs, except *NtbZIP04*, which was not expressed in the leaf or stem. *NtbZIP69* genes was more highly expressed in the calyx than in other organs. *NtbZIP29* and *NtbZIP69* were highly expressed in petal tissue, and *NtbZIP70* was highly expressed in anther tissue. *NtbZIP01* was more highly expressed in both stigma and leaf tissue than in other tissues, and its expression in these tissues was higher than other *NtbZIP*s; similarly *NtbZIP49* was more highly expressed in stem, fruit, and root tissue than other genes. In addition, we analysed whether the expression patterns of the 11 *NtbZIP*s in the 8 organs were correlated (Fig. 6b). This analysis found that the expression of different genes in the plant organs were significantly correlated; some genes were positively correlated (*NtbZIP49* with *NtbZIP37, NtbZIP69,* and *NtbZIP70; NtbZIP01* with *NtbZIP04, NtbZIP76,* and *NtbZIP18*), whilst some were negatively correlated (*NtbZIP37* and *NtbZIP29*; *NtbZIP70* with *NtbZIP01, NtbZIP18, NtbZIP29, NtbZIP76*). All of the 11 *NtbZIPs* showed positive correlation with different tissues (Additional file 5: Figure S1). For example, the *NtbZIP70*, *NtbZIP74*, *NtbZIP29* showed highly positive correlation with the anthers, the stem and the petals, respectively. However, *NtbZIP63* showed negative correlation with anthers.

### Expression patterns of *NtbZIP*s in response to different stresses

**Fig. 7:**
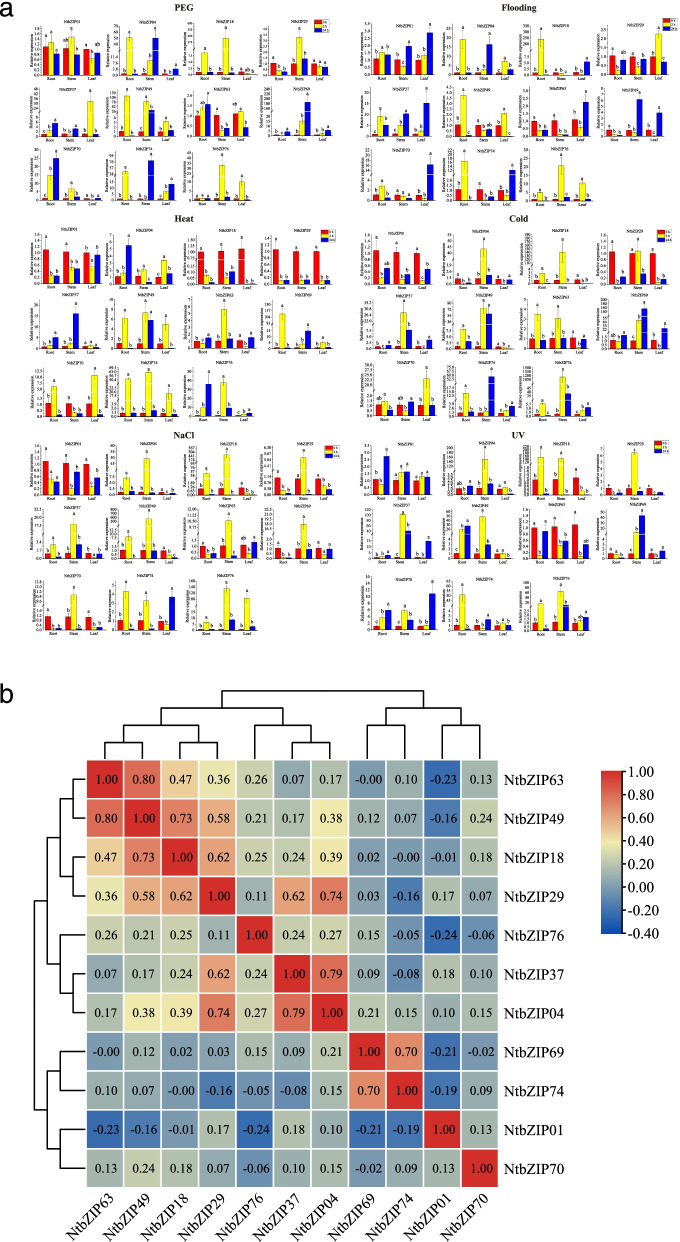
Expression of 11 *Nicotiana tabacum* L. *bZIP*s in plants subjected to abiotic stresses (strong UV radiation, flooding, PEG, NaCl, and hot and cold treatments) at the seedling stage. bZIP: basic leucine zipper; qRT-PCR: Real-time quantitative PCR. **a** Expression patterns of 11 *Nicotiana tabacum* L. *bZIP*s in leaf, root, and stem organs were examined by qRT-PCR. Error bars represent standard error. Lowercase letters above bars indicate a significant difference (*P* < 0.05, LSD) among the treatments. bZIP: basic leucine zipper; qRT-PCR: Real-time quantitative PCR. **b** Positive number: positively correlated; negative number: negatively correlated. Red numbers indicate a significant correlation at the 0.05 level

To study the expression of *NtbZIP*s under abiotic stress, we used qRT-PCR to analyse the expression of 11 *NtbZIP*s in roots, stems, and leaves under 6 abiotic stresses: polyethylene glycol (PEG), flooding, heat, cold, NaCl, and strong ultraviolet (UV) radiation treatments (Fig. 7a). The expression patterns of *NtbZIP*s differed in response to the 6 abiotic stresses, with some *NtbZIP*s either significantly induced or inhibited. In the early stages of stress treatments, the expression patterns of most genes changed significantly (Fig. 7a); in response to different stress treatments, the expression of some *NtbZIP*s also changed with time or in different organs. For example, in response to PEG stress, *NtbZIP04, NtbZIP18*, and *NtbZIP49* were first significantly upregulated in root tissue, and then downregulated; *NtbZIP49* also demonstrated this pattern of expression in stem and leaf tissue. In response to heat stress, *NtbZIP18* and *NtbZIP29* were significantly downregulated in the root, stem, and leaf, whereas *NtbZIP74* was first significantly upregulated, and then downregulated in these tissues. Some other genes showed changes in specific organs: for example, the response of *NtbZIP76* to cold stress was significant only in the stem. In addition, we analysed whether there were correlations between the expression patterns of these *NtbZIP*s (Fig. 7b), detecting positive correlations among most *NtbZIP*s*. NtbZIP49* and *NtbZIP63* were significantly positively correlated, and 4 genes (*NtbZIP04*, *NtbZIP18*, *NtbZIP37* and *NtbZIP49*) were significantly positively correlated with *NtbZIP29.* Under different abiotic stresses, plant tissues showed different correlations with gene expression patterns (Additional file 6: Figure S2). Under flooding stress, leaf tissue showed significant positive correlations with *NtbZIP69, but a negetive correlation with NtbZIP29.* Similarly, under heat stress, root tissue was significantly positively correlated with *NtbZIP29*, *NtbZIP18* and *NtbZIP01*, but significantly negatively correlated with *NtbZIP04* and *NtbZIP76.* Furthermore, The number of genes that positively related to stem tissue were more than that of leaf and root tissue under 6 abiotic stresses.

## Discussion

Tobacco is an important commercial crop and model plant. The bZIP family is one of the most important TF families found in the plant kingdom. Increasing evidence suggests that bZIP TFs play important roles in regulating growth and development, and responses to biotic and abiotic stresses [3, 15, 52]. Previous research on the functions of bZIP TFs has mainly focused on *A. thaliana,* and there were also relevant studies in other crops, vegetables, and fruits [4, 8, 9, 28, 42, 53]. Compared with other important plants, there has been little research on the bZIP family in tobacco; therefore, we systematically analysed the *bZIP* family in tobacco*.*

In this study, 77 *bZIP*s were identified from tobacco (3734 Mb) on the basis of a constructed phylogenetic tree. Previous studies have identified 75 *bZIPs* in *A. thaliana* (117 Mb) [8], 89 in rice (466 Mb) [4], 125 in maize (2182 Mb) [54, 55], 160 in soybean [56] (915 Mb), 55 in grape [53] (490 Mb), and 96 in tartary buckwheat [44] (489 Mb). Results from this study suggest that the number of bZIPs in tobacco is similar to that found in *A. thaliana*, more than in grape and in *Carthamus tinctorius*, and fewer than in rice and soybean. There is no correlation between the number of *bZIP*s in a species and genome size. According to cluster analysis and the similarity of protein sequences with *A. thaliana*, the 77 *NtbZIP*s divided into 11 subfamilies, and 2 subfamilies (J and K) were newly identified. The number of subfamilies was consist with the buckwheat [44], caster bean [57], more than *A. thaliana* [8] and grape [53], lower than Chinese jujube [45] and poplar [46]. The J subfamily is consistent with grapevine [53] and Chinese jujube [45], whereas the K subfamily is the same as that in soybean [56] and radish [27]. Subfamilies A and S had the largest number of *bZIP*s, similar to as seen in *A. thaliana* [8]. Four of the *NtbZIP*s did not have obvious clusters; this also occurred in grape [53], and banana [51]. The H subfamily did not contain any tobacco genes, which suggested that the H subfamily was lost in tobacco.

Gene replication, including tandem, segment, and whole genome replication, plays an important role in the evolution of organisms and the expansion of gene family [58]. In this study, 16 pairs of segmental duplications were observed in the *NtbZIP* genes, without any tandem replications, which also occurred in poplar [46]. These results suggest that segmental replication may contribute more than tandem duplications to the expansion of the bZIP family in tobacco, consistent with previous studies in other species, including rice [4], maize [59], grape [60], and cucumber [61]. The number of bZIPs in tobacco is similar to that found in *A. thaliana* [8], more than in grape [53] and in *Carthamus tinctorius* [43] and fewer than in rice [8] and soybean [56]. This difference may be caused by the whole-genome duplication event that occurred after the earliest ancestors of the other species diverged [62]. These segment duplication events may be promote the occurrence and evolution of some *NtbZIP* genes, which also occurred in *Populus trichocarpa* [63] and *Sorghum bicolor* [62]. From the results of chromosome distribution, there were no *NtbZIP* genes on Chr8 and Chr14, illustrating that the *NtbZIP* gene family may be affected by gene deletion during the process of evolution [53]*.* Therefore, some *bZIP* genes may be caused by some replication events, which further illustrates that replication events play an important role in the rapid expansion of *bZIP* family members in plants.

In addition, the phylogenetic analysis was confirmed by gene structure and conserved motif analysis of the 77 *bZIP*s. Analysis of the gene structure of the *NtbZIP* family identified several differences among the *NtbZIP*s. We determined that the number of introns present varied from 0 to 17, with 16.88% of the genes containing no introns, similar to as seen in rice [8], soybean [56], and sorghum [64]. The two duplicated genes tended to cluster in a subfamily, and previous studies have also shown such results [64]. The results of phylogenetic analysis confirmed this, with *NtbZIP30* and *NtbZIP77* (1 exon and 0 introns), *NtbZIP11* and *NtbZIP75* (3 exons and 2 introns), and *NtbZIP21* and *NtbZIP59* (2 exons and 1 intron) clustered to subfamilies S, A, and F, respectively. The subfamilies G, J, and K contained genes with more than 9 introns, while other subfamilies had genes with fewer introns. Nuruzzaman et al. [65] studied the bZIP TF family in rice, and found that the rate of intron acquisition was slower than the rate of intron deletion after segmental duplication. Therefore, we can infer that the G, J, and K subfamilies may contain primitive genes. In most gene families, the increase and loss of introns and exons is one of the mechanisms for diversification [66], and this was also observed here. For example, *NtbZIP47* had 1 intron and 2 exons, while its paralogous gene *NtbZIP45* contained 0 introns and 1 exon, indicating the loss of an exon during evolution. A similar phenomenon also occurred in the *bZIP* gene families of apple [67] and soybean [56]. The increase of introns is conducive to the adaptation of plants to the environment, which is beneficial to evolution. However, genes with fewer introns may be conducive to rapid response to the environment [68]. In this study, a total of 10 motifs were identified in the 77 NtbZIP proteins. Although different subfamilies have different motifs and the motif encoding bZIP is conservative. Most members contained the motif 1 conserved domain, which contains an N- × 7-R/K conserved sequence, indicating conservation of the bZIP TF family in tobacco. Furthermore, analysis of the conserved motifs indicated that each subfamily member shared similar motifs, which were the same as those in buckwheat [27, 44]. For example, all NtbZIP proteins shares motifs 1, 3 and 5 in group I. Both Group S and I contain motifs 1 and 3. These results not only support the classification of the subfamilies, but also indicate that members in the same subfamily have similar evolutionary histories and physiological functions [50].

The *bZIP* family plays an important regulatory role in growth and development, including the development of flowers and fruits [51, 69, 70]. Analysis of gene expression profiles can provide an important basis for understanding the potential biological functions of genes. The functions of bZIP TFs in tobacco still need to be further studied. Here, we selected 11 *NtbZIP*s, clustered in different subfamilies and with significant differences, to study the expression patterns in different organs. All of the *NtbZIP* genes investigated were expressed in at least 1 of the organs tested, indicating that they may play a broad role in the growth and development of tobacco, similar to as the former reported research in soybean [62] and buckwheat [71]. Most genes were expressed in all organs, consistent with previous studies [8, 43, 45, 72], however there were some genes that were not expressed in some of the organs, as previously seen in studies of grape, soybean, and apple [53, 56, 67]. For instance, the NtbZIP04 gene was not expressed in leaf and stem. In this study, according to the phylogenetic tree constructed by *NtbZIP* genes homologous to *AtbZIP* genes (Fig. 1), the function of *NtbZIP* gene homologous to *AtbZIP* can be predicted. Clarifying the function of these *NtbZIP* genes can lay a foundation for the growth and development of tobacco and other species in future. As the reproductive organs of plants, flowers and fruits are the main structures of all angiosperms [73]. In comparison to non-reproductive tissues (root, stem, and leaf), the expression levels of *NtbZIP29* was significantly higher in the petal, stigma and anther tissues. *AtbZIP18*, a gene in *A. thaliana* homologous to the tobacco gene *NtbZIP29.* The study of *AtbZIP18* gene in *A. thaliana* showed that *AtbZIP18* had high expression in pollen and played an important role in male gametophyte [72]. Therefore, we speculated that *NtbZIP29* is involved in the development of flowers and fruits. *NtbZIP01* showed high expression in leaves. *AtbZIP41*, a gene homologous to *NtbZIP01*, has been proven to recognise and specifically bind with the G-box to form the G-box-binding factor 1/bZIP41 binding factor, which is constitutively expressed in *A. thaliana* leaves [74–76]. According to the subfamily classification result (Fig. 1), *NtbZIP01* belongs to G subfamily that contains the G-box-binding factor. The specific expression of *NtbZIP01* gene in leaf may be due to its special structure. The expression level of *NtbZIP04* was highest in stigma; the *A. thaliana* genes homologous to *NtbZIP04*, *AtbZIP34* and *AtbZIP61*, have been confirmed in several studies to interact with other bZIP TFs to participate in plant development [48, 74, 77]. The *NtbZIP04* gene may be has similar function with the homologous genes of *AtbZIP34* and *AtbZIP61.*

*bZIP* genes play an vital role in plant stress response, which have illustrated in previous studies [4, 8, 9]. We also studied the gene expression patterns of these 11 *NtbZIP*s under 6 abiotic stresses. The results showed that the expression of most of the *NtbZIP*s were significantly different under abiotic stresses, which was consistent with previous studies [28, 53, 78]. For example, under drought stress, *NtbZIP*s were upregulated in the root (9), stem (9), and leaf (7). There were 8, 5, 11 *NtbZIP*s upregulated in the root, stem, and leaf, respectively. Additionally, the same gene showed opposite expression patterns under different stress. *NtbZIP04* gene was up-regulated in leaves under heat stress, but down-regulated under cold stress. These results illustrate that *bZIP* genes are widely involved in the response to abiotic stress and bZIP TFs participate in a complex cross regulatory network [64]. *NtbZIP*49 and *NtbZIP76* responded to PEG, flooding, heat, cold, and NaCl abiotic stresses at the same time, indicating that these two genes are co-expressesed under a variety of adverse conditions, which was consistent with the study of *Sorghum bicolor* [64]. We found high expression of *NtbZIP18* under all 6 abiotic stresses. *NtbZIP18* is homologous with subfamily F members *AtbZIP19*, *AtbZIP23,* and *AtbZIP24* of *A. thaliana*. Previous studies showed that *AtbZIP24* is an important regulator of the salt stress response in plants, and regulates complex transcription networks involved in abiotic stress resistance [79, 80]. The *NtbZIP18* gene may have similar functions to *AtbZIP24* gene. Subsequent studies can verify the function of *NtbZIP18* gene to prove whether it can be used to improve the resistance of varieties to various abiotic stress. *NtbZIP49* showed the highest expression levels in stem tissue under NaCl stress; this gene is homologous with *AtbZIP75* of *A. thaliana*, reported to respond to salt stress [81]. Among the six abiotic stress, *NtbZIP74* showed high expression in root. *NtbZIP74* gene and *AtbZIP67* are homologous genes and both come from subfamily A that is mainly related to abscisic acid or some stress signal pathways [82]. Previous confirmed that *AtbZIP67* played a positive role in ABA-induced AHT1 expression. The up-regulated expression of *NtbZIP74* gene in roots may be due to its participation in ABA signaling pathway. In this analysis, *NtbZIP76* showed significant responses to heat stress. This gene is homologous to *AtbZIP17* (*A. thaliana*), which plays a vital role in the endoplasmic reticulum heat stress pathway, supporting our findings in tobacco [83,84]. From the expression heat map, we found that most of the *NtbZIP*s were positively correlated*.* By considering the interactions between the expression patterns and complex proteins, we could find a feedback mechanism network to coordinate the expression of multiple genes.

## Conclusion

In summary, we analysed the whole genome of the *bZIP* family in tobacco, including gene structure, chromosomal location, sequence homology, synteny, and expression patterns. In this study, a total of 77 *NtbZIP*s were identified and divided into 11 subfamilies, providing preliminary information for the characterisation of *NtbZIP* function. Although there were many types of proteins, the exon–intron structure of genes, and the motif arrangement, of bZIP proteins from the same subfamily, supported the classification predicted by the phylogenetic tree. The 77 *NtbZIP*s were unevenly distributed across 22 tobacco chromosomes. A total of 16 pairs of segmental duplication were identified, illustrating the important role of gene duplication in the expansion of the *NtbZIP* family. In addition, expression analysis of *NtbZIP*s in different tissues in response to 6 abiotic stresses implied that *NtbZIP*s participate in the development of tobacco. *NtbZIP49* may be important in the development of flowers and fruits; *NtbZIP18* is an important regulator in abiotic stress. These results will be helpful for the further study of the physiological processes performed by the *bZIP* family in tobacco.

## Methods

### Identification of bZIP family in tobacco

The entire genome sequence of *Nicotiana tabacum* L. was downloaded from the solgenomics website (https://solgenomics.net/organism/Nicotiana_tabacum/genome). Two BLASTp methods were used to obtain *NtbZIP*s [85, 86]. Firstly, all *AtbZIPs* were used to search for possible *NtbZIP*s from the tobacco genome by using BLASTp (score value ≥ 100 and E value ≤ 1e^−10^). Secondly, the Hidden Markov Model file of the bZIP domain was downloaded from the Pfam protein family database (http://pfam.xfam.org) [87]. Both HMMER 3.0 (http://hmmer.org/), with a cutoff of 0.01, and SMART (Simple Modular Architecture Research Tool) (https://academic.oup.com/nar/article/49/D1/D458/5940513), were used to identify and verify *bZIPs.* Furthermore, we analysed the basic characteristics of the identified bZIP proteins using the ExPASY website (https://web.expasy.org/), including the CDS, pI, and Mw.

### Exon/intron structures and conserved motif analysis

ClustalW was used to align the default parameters of the multiple amino acid sequences of the bZIPs identified [88]. GeneDoc software (https://genedoc.software.informer.com/) was used to manually adjust the deduced amino acid sequences in the bZIP domain. The exon/intron composition of the *bZIP*s was analysed by the Gene Structure Display Server (GSDS: http://GSDS.cbi.pku.edu.cn) [89]. MEME (http://meme-suite.org/tools/meme) was used to analyse the motifs of the bZIP proteins [90, 91]. Optimisation parameters were set as follows: repetition times, arbitrary; maximum number of motifs, 10; optimal width of each motif, between 6 and 200 residues [86, 91, 92].

### Chromosomal distribution, gene duplication and collinear analysis with other species

All *NtbZIP*s were mapped to tobacco chromosomes using Circos [93], according to the physical location information in the tobacco genome database. We used the default parameters of the Multiple Collinearity Scan toolkit (MCScanX) to detect gene duplication [94]. Homology between *bZIP*s in tobacco and the other 7 species (*A. thaliana*, *O. sativa*, *F. tataricum*, *S. lycopersicum*, *H. vulgare*, *V. vinifera* and *S. tuberosum*) was analysed through the Dual Synteny Plotter software (https://github.com/CJ-Chen/TBtools).

### Phylogenetic analysis and classification of the *NtbZIP* family

All identified *NtbZIP*s were divided into several subfamilies, according to the classification of the *AtbZIP* family. Using the neighbour-joining method of Molecular Evolutionary Genetics Analysis (MEGA) X, the phylogenetic tree was built using Geneious R11 with a BLOSUM62 matrix, using the Jukes Cantor substitution model, a global alignment with free end gaps, and a bootstrap value of 1000. The full-length amino acid sequences of bZIP proteins were compared with the newly identified *NtbZIPs* for phylogenetic analysis (Additional file 1: Table S1), derived from *A. thaliana*, *O. sativa*, *F. tataricum*, *S. lycopersicum*, *H. vulgare*, *V. vinifera* and *S. tuberosum* (Uniprot: https://www.uniprot.org/).

### Plant materials, growth conditions, and abiotic stress in tobacco

‘K326’, which is a typical, cultivated, common tobacco variety with good yield and quality, was used in this study. ‘K326’ has been grown in the greenhouse of the Guizhou University tobacco research base since 2020. The seeds of ‘K326’ were sown in a special substrate for flue-cured tobacco and raised by floating seedling in a growth room with a 16 h/25 °C day and 8 h/20 °C night regime, and a relative humidity of 75%. We collected the calyx, petal, anther, stigma, leaf, stem, fruit, and root separately from 3 plants showing good and similar growth, 71 days after transplanting, and repeated sampling for three times. To investigate the expression patterns in response to different abiotic stresses, 11 *bZIP*s were selected for further study. Tobacco plants were subjected to the following abiotic stresses 63 days post-sowing: salt (5% NaCl), water flooding (whole plant), drought (20% PEG6000), UV exposure (70 µW/cm^2^, 220 V, 30 W), high temperature (38 °C), and low temperature (4 °C). Every abiotic stress treatment was performed with 3 replicates; materials were sampled for qRT-PCR analysis and enzyme activity determination after treatment for 2 h and 24 h. Collected samples were kept at -80 °C until analysis.

### Total RNA extraction, cDNA reverse transcription, and qRT-PCR

The RNAprep Pure Plant Kit (TIANGEN, DP432) was used to extract total RNA from samples. The cDNA was generated using the FastKing gDNA Dispelling RT SuperMix kit (TIANGEN, KR118), following manufacturer’s instructions. We selected 11 *NtbZIP*s that all with close evolutionary relationships to *AtbZIP*s from different subfamilies to study gene expression patterns. The qRT-PCR primers used to analyse gene expression were designed by Primer Premier 6 (http://www.premierbiosoft.com/) (Additional file 7: Table S5). We used the *Actin* gene as an internal reference, as it is stably expressed at each growth stage in almost all tissues. The qRT-PCR analysis, performed with Talent qPCR PreMix (SYBR Green) (TIANGEN, FP209), was repeated at least 3 times and the 2^−ΔΔCt^ method was used to caculate the final test results [95].

### Statistical analysis

DPS (Data Processing System) software was used to analyse the variance of the data, and data were compared with the least significant difference test at the 0.05 and 0.01 levels. OriginPro 2016 software (OriginLab, https://www.originlab.com/) was used for the generation of graphs.

## Supplementary Information

**Additional file 1:** **Table S1.** List of the 77 *Nicotianatabacum *L.* bZIP*s identified in this study. bZIP: basic leucine zipper.

**Additional file 2:** **Table S2.** Analysis and distribution of the conserved motifs in *Nicotiana tabacum *L bZIP proteins. bZIP: basic leucine zipper.

**Additional file 3:** **Table S3.** The 16 pairs of segmental duplications in *Nicotiana tabacum *L. *bZIP*s.bZIP: basic leucine zipper.

**Additional file 4:** **Table S4.** One-to-one orthologous gene relationships between *Nicotiana tabacum *L. and other plants.

**Additional file 5:** **Figure S1.** The correlation analysis between 8 different tissues and expression patterns of the 11 *Nicotiana tabacum *L. *bZIP *genes.

**Additional file 6:** **Figure S2.** The correlation analysis between 3 different tissues and expression patterns of the 11 *Nicotiana tabacum* L. *bZIP* genes under abiotic stresses.

**Additional file 7:** **Table S5.** Primer sequences used for real-time quantitative PCR.

## Data Availability

The whole *Nicotiana tabacum* genome sequence information was obtained from the solgenomics website (https://solgenomics.net/organism/Nicotiana_tabacum/genome) and the website is open to all researchers. The tobacco materials (‘K326’) used in this study were supplied by Prof. Liu Renxiang of Guizhou University. The datasets supporting the conclusions of this article are included in the article and its Additional files.

## Abbreviations

bZIP: Basic leucine zipper
CDS: Coding sequence length
DPS: Data Processing System
LG: Linkage group
MEGA: Molecular Evolutionary Genetics Analysis
MEME: Multiple Expectation maximizations for Motif Elicitation
Mw: Protein molecular weight
PEG: Polyethylene glycol
pI: Isoelectric point
qRT-PCR: Real-time quantitative PCR
SMART: Simple Modular Architecture Research Tool
TF: Transcription factor
UV: Ultraviolet
W: Predicted Subcellular location
