# Supplementary figures and images for: Genome-wide identification and expression analysis of the bZIP transcription factor family genes in response to abiotic stress in Nicotiana tabacum L
Source: BMC Genomics. 2022 Apr 22;23:318. doi: 10.1186/s12864-022-08547-z (PMC9027840; doi:10.1186/s12864-022-08547-z)

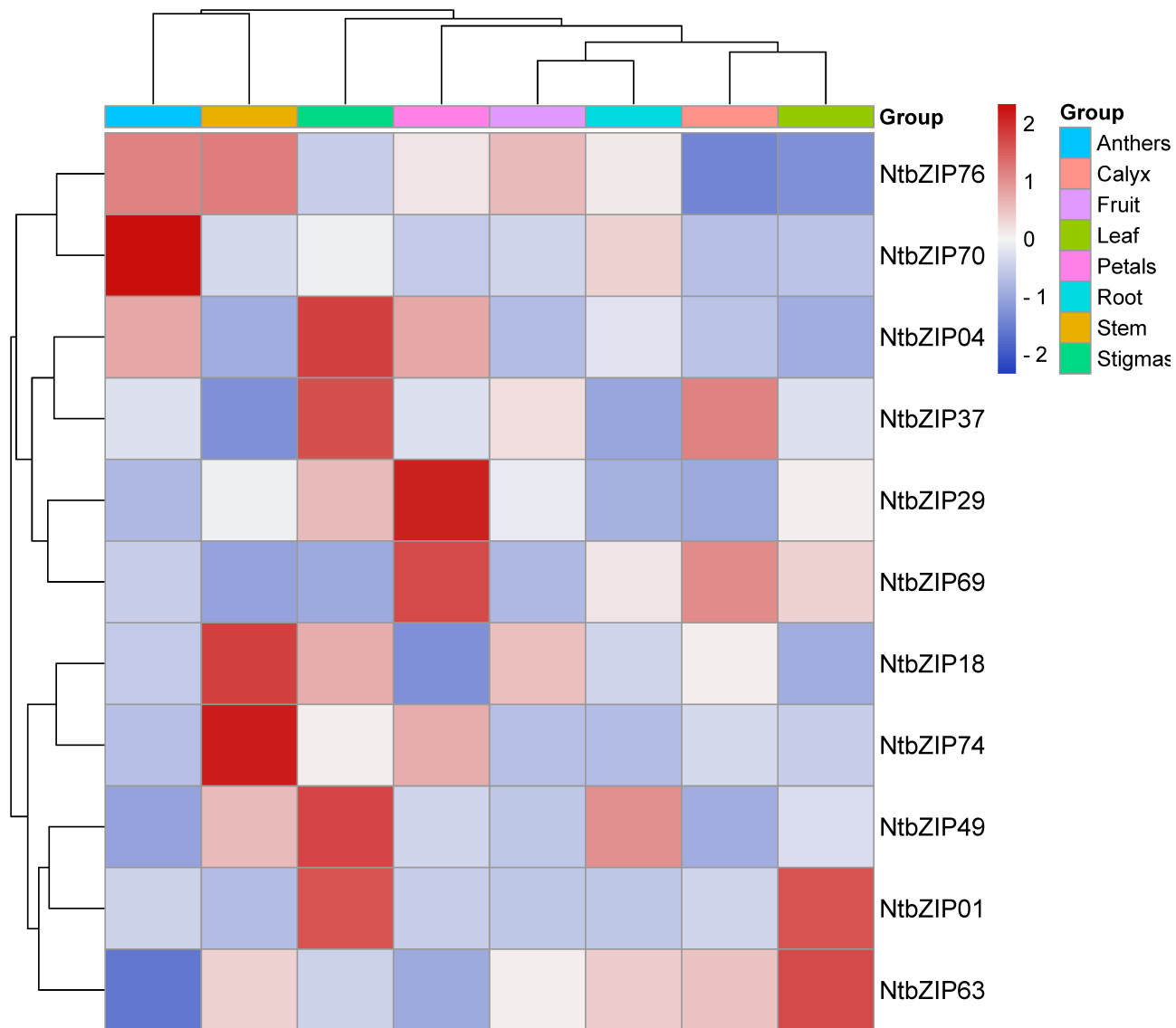

Supplement: Supplementary file 1 — Additional file 1: Table S1. List of the 77 Nicotianatabacum L. bZIPs identified in this study. bZIP: basic leucine zipper. [file 12864_2022_8547_MOESM1_ESM.pdf]

## PEG

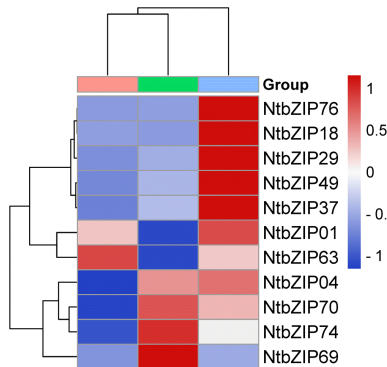

## Flooding

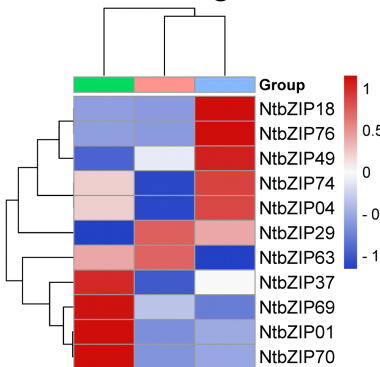

## Heat

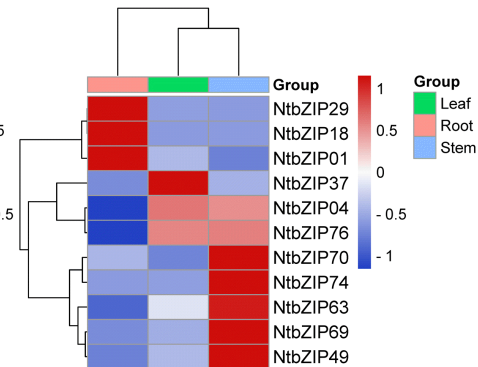

## Cold

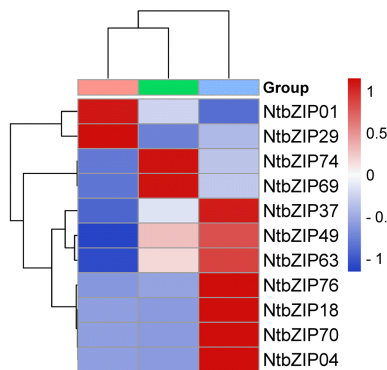

## NaCl

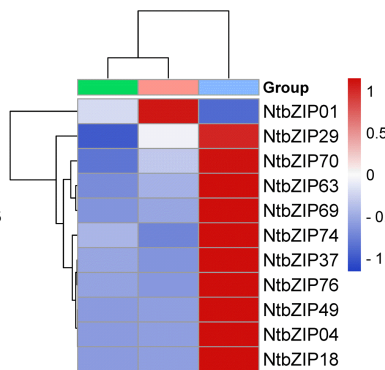

## UV

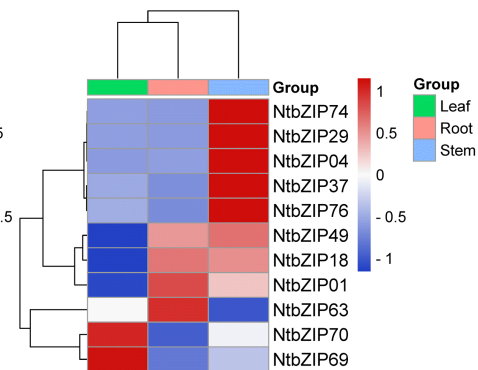

Supplement: Supplementary file 2 — Additional file 2: Table S2. Analysis and distribution of the conserved motifs in Nicotiana tabacum L bZIP proteins. bZIP: basic leucine zipper. [file 12864_2022_8547_MOESM2_ESM.pdf]
